# Supplementary material for: Role of matrix metalloproteinase-9 in neurodevelopmental deficits and experience-dependent plasticity in Xenopus laevis
Source: eLife. 2021 Jul 20;10:e62147. doi: 10.7554/eLife.62147 (PMC8315794; doi:10.7554/eLife.62147)
Supplement: Supplementary file 1. — Input resistance and membrane capacitance values showed no significant differences between experimental groups. [file elife-62147-supp1.docx]

**Supplementary Table 1. Intrinsic cellular properties in different experimental groups.** Input resistance and membrane capacitance values showed no significant differences between experimental groups.

| **Figure** | **Experiment** | **Group** | **Sample size** | **Input resistance in MΩ (Mean ± SD)** | **Membrane Capacitance (Mean ± SD)** |
| --- | --- | --- | --- | --- | --- |
| 1 | MMP9 overexpression | MMP9 transfected | 15 | 798.08 ± 835.23 | 15.79 ± 5.6 |
| 1 | MMP9 overexpression | MMP9 nontransfected | 19 | 1099.9789 ± 333.57 | 9.0 ± 4.02 |
| 1 | MMP9 overexpression | GFP control transfected | 11 | 1305.5091 ± 655.88 | 10.47 ± 5.06 |
| 1 | MMP9 overexpression | GFP control nontransfected | 14 | 1183.6857 ± 815.88 | 9.12 ± 2.69 |
| 1 | MMP9 overexpression | Control | 22 | 993.0682 ± 308.16 | 10.16 ± 2.63 |
|  |  |  |  |  |  |
| 2 | Pharmacological inhibition of MMP9 | Control | 22 | 1156±337 | 10.49±3.4 |
| 2 | Pharmacological inhibition of MMP9 | VPA | 22 | 1132±356 | 13.35±3.8 |
| 2 | Pharmacological inhibition of MMP9 | VPA + SB-3CT | 25 | 1080±340 | 14.54±5.6 |
| 2 | Pharmacological inhibition of MMP9 | SB3-CT | 26 | 1286±526 | 10.33±3.5 |
|  |  |  |  |  |  |
| 3 | MMP9 morpholino | VPA+Control MO | 9 | 1083±314 | 11.23±1.3 |
| 3 | MMP9 morpholino | VPA+MMP9 MO | 10 | 1460±114 | 10.04±2 |
| 3 | MMP9 morpholino | Control MO | 28 | 1086±440 | 12.52±4.1 |
| 3 | MMP9 morpholino | MMP9 MO | 14 | 1154±456 | 11.35±3.3 |
